# Pathogenicity virulence of *Beauveria* spp. and biosafety of the BbMQ strain on adult ectoparasitic beetles, *Dastarcus helophoroides* Fairmaire (Coleoptera: Colydiidae)

**DOI:** 10.3389/fvets.2023.1077473

**Published:** 2023-05-16

**Authors:** Youjun Zhou, Ciding Lu, Zhenghao Chen, Shuting Ye, Xinyuan Fang, Zhuhe Zhang, Shouping Cai, Feiping Zhang, Guanghong Liang

**Affiliations:** ^1^Forestry College, Fujian Agriculture and Forestry University, Fuzhou, Fujian, China; ^2^Forest Pest and Disease Control and Quarantine Station, Forestry Bureau of Fuzhou City, Fuzhou, Fujian, China; ^3^Fujian Academy of Forestry, Fuzhou, Fujian, China; ^4^Provincial Key Laboratory of Integrated Pest Management in Ecological Forests, Fujian Agriculture and Forestry University, Fuzhou, Fujian, China

**Keywords:** *Dastarcus helophoroides* Fairmaire, *Beauveria bassiana*, *Monochamus alternatus* Hope, pathogenicity, biological control, virulence

## Abstract

**Introduction:**

*Beauveria spp*. and *Dastarcus helophoroides* Fairmaire adults were simultaneously released to attack elder larvae or pupae of *Monochamus alternatus* in pine forests in China. However, little is known about the pathogenicity virulence and biosafety of *Beauveria* spp. on beneficial adults of *D. helophoroides*, and specific *Beauveria bassiana* (Bb) strains should be selected for synthetic release together with *D. helophoroides*.

**Methods:**

A total of 17 strains of *Beauveria* spp. were collected, isolated, and purified, and then their mortality, cadaver rate, LT50, spore production, spore germination rate, and growth rate of *D. helophoroide* adults were calculated based on 0–20 days data after spore suspension and powder contact.

**Results and discussion:**

The lethality rate of BbMQ, BbFD, and BbMH-03 strains to *D. helophoroides* exceeded 50%, and the cadaver rate reached 70.6%, among which the mortality rate (82.22%), cadaver rate (47.78%), spore production (1.32 × 10^9^ spores/ml), spore germination rate (94.71%), colony dimension (49.15 mm^2^), and LT_50_ (10.62 d) of the BbMQ strain were significantly higher than those of other strains (*P* < 0.01), and the mortality of *D. helophoroides* adults increased significantly with increased spore suspension concentration, with the highest mortality reaching 92.22%. This strain was identified as *Beauveria bassiana* by morphological and molecular methods, while the BbWYS strain had a minimum lethality of only 5.56%, which was safer compared to other strains of adult *D. helophoroide*. Consequently, the biological characteristics and pathogenicity of different *Beauveria bassiana* strains varied significantly in their effects on *D. helophoroide* adults, and the safety of different strains should be assessed when they are released or sprayed to control multiple pests in the forest. The BbMQ strain should not be simultaneously sprayed with releasing *D. helophoroide* adults in the same forest, while the BbWYS strain can be used in concert with *D. helophoroide* to synergize their effect.

## 1. Introduction

*Beauveria* spp. contains a variety of insect pathogenic fungi comprising one of the most important natural enemies and components of integrated pest management (IPM) for agricultural pests, attracting much attention and widely used in application efforts worldwide (1, 2). Recently, they were commonly used to control some important defoliators and longhorn beetles in China such as *Dendrolimus punctatus* Walker (3, 4), *D. houi* Lajonquiere (5) and *Monochamus alternatus* Hope (6), achieving significant control effects (5, 7, 8) and becoming popular biological pesticides. Meanwhile, *M. alternatus*, as the main vector of pinewood nematode (*Bursaphelenchus xylophilus*) and the causal agent of pine wilt disease in China, directly or indirectly kills hundreds of millions of pine trees in 19 provinces (9, 10). *Dastarcus helophoroides* Fairmaire, a dominant endoparasitic beetle, which also has been released to kill *M. alternatus* in the forests achieved good levels of control (6, 11–15).

Earlier research revealed that *D. punctatus* and *M. alternatus* have overlapping life cycles, and they annually coexist from March to May in the same *P. massoniana* forests (16, 17). Coincidentally, *Beauveria* spp. was sprayed to suppress the larvae of *D. punctatus* during this period to optimize its effect due to optimal temperature and humidity in the forests, and the infected caterpillars of *D. punctatus* will probably provide the pathogen for their next generation. Meanwhile, it is also the best period for the mass release of *D. helophoroides* adults to kill the old larvae or pupae of *M. alternatus* (15). Obviously, these two natural enemies will inevitably coexist in the same forest. What will happen if *D. helophoroides* adults encounter *Beauveria* spp.? Could it infect *D. helophoroides* and thus affect its population stability and pest control effectiveness? To date, except for a few reports of *Beauveria bassiana* (Bb) strain having very low mortality in *D. helophoroides* adults (18), little is known about pathogenicity and biosafety of *Beauveria* spp. on *D. helophoroides*. Consequently, 17 strains of *Beauveria* spp. were collected, isolated, purified, and inoculated on *D. helophoroides* adults to screen and obtain the highest and lowest virulence strains for practice use and biosafety evaluation (19).

## 2. Methods and materials

### 2.1. Insect acquisition, rearing, and screening methods

The 20-day-old adults of *D. helophoroides* were purchased from the Southern Natural Enemy Breeding and Application Engineering Technology Research Center, the State Forestry Administration (Changsha, Hunan, China), and stored in the refrigerator under 4°C at Fujian Agriculture and Forestry University (Fuzhou, Fujian, China). Prior to experiments, 30 healthy *D. helophoroides* adults were taken out and put into a modified plastic box (16 × 10.5 × 5 cm, L × W × H) under room temperature (25 ± 1°C), allowed 5 min to recover, and then transferred to an artificial climate chamber (Shanghai Yiheng, MGC-300H, 20L) under 25 ± 1°C, L:D = 12:12 h, RH = 70 ± 10% for 2 d. All samples were screened by removing dead and dying individuals in the lab during the process of mass rearing, and three replicates were prepared.

The plastic box was modified to be a rearing box for *D. helophoroides* adults by making 15 ventilation holes (d = 1 mm) by using a soldering iron and placing two paper towels (15 × 5 cm, length × width) on the bottom of the rearing box. In total, five pieces of *P. massoniana* bark and one section of *P. massoniana* wood (d = 4 cm, h = 6 cm) were also placed on the paper towels to simulate habitat. Finally, a centrifuge tube (3 ml) containing an artificial diet for *D. helophoroides* and a moist cotton ball was placed to provide food and water, respectively.

Formulation of an artificial diet for *D. helophoroides* adults was as follows: 20 g larval powder of *M. alternatus* larvae, 60 g powder of *Zophobas morio*, 20 g powder of *Tenebrio molitor*, 10 g powder of *Antheraea pernyi* pupae, 10 g dried yeast, 10 g glucose, 10 g lyophilized yolk powder, 50 g pine powder (containing 15 g epidermal powder plus 35 g xylem powder), and 20 g sunflower oil, with all ingredients thoroughly mixed (20). The prepared artificial diet was put into centrifuge tubes (3 ml) and stored in the refrigerator at −4°C.

### 2.2. Collection, isolation, and purification of tested strains

Most strains of *Beauveria* spp. were naturally collected from infected insect cadavers in the forests in Fujian, China, and a few strains were obtained from the Fujian Academy of Forestry (FAF, Fujian, China). The PDA medium was used to obtain pure cultures after delineation, and by continuous isolation in a sterile ultra-clean bench in the lab. A total of 17 strains of *Beauveria* spp. were obtained and identified based on the fungal morphological characteristics (Table 1) and stored at 4°C in the refrigerator.

**Table 1:** All strains of *Beauveria* spp. for this experiment in Fujian, China.

**Strain number**	**Host insects**	**Collecting place**	**Year**
BbHA-03	*Dendrolimus* sp. pupae	Huian, Fujian, China	2016
BbSX-06	*D. punctatus* larvae	Shaxian, Fujian, China	2015
BbYT-03	*D. houi* adult	Yongtai, Fujian, China	2018
BbXP	*D. houi* larvae	Xiapu, Fujian, China	2020
BbMH-03	*D. houi* larvae	Minhou, Fujian, China	2017
BbYT-02	*D. houi* larvae	Yongtai, Fujian, China	2017
BbMH-04	*D. houi* larvae	Minhou, Fujian, China	2018
BbDH	*Cerambycidae* sp. larvae	Dehua, Fujian, China	2020
BbWYS	*Membracidae* sp.	Mount Wuyi, Fujian, China	2017
BbAX-04	*Porthesia similis* larvae	Anxi, Fujian, China	2019
BbQZ-03	*M. alternatus* larvae	Quanzhou, Fujian, China	2016
BbMH-02	*D. houi* larvae	Minhou, Fujian, China	2017
BbFD	*D. houi* pupae	Fuding, Fujian, China	2020
BbMH-01	*D. houi* larvae	Minhou, Fujian, China	2017
BbLJ-06	*M. alternatus* larvae	Lianjiang, Fujian, China	2014
BbMQ	*D. houi* larvae	Minqing, Fujian, China	2018
BbQZ-08	*D. punctatus* larvae	Quanzhou, Fujian, China	2018

### 2.3. The virulence of *Beauveria* spp. spore suspension on *D. helophoroides* adults

The 17 strains were inoculated in line on sterile Petri dishes (d = 10 cm) containing the PDA medium and incubated purely in an artificial incubator (Shanghai Yiheng, LRH-150) under 26 ± 1°C, 80 ± 10% humidity for 14 d; then each strain after full sporulation was eluted with sterile water containing 0.01% Tween-80 in a 50 ml centrifuge tube and fixed to 10 ml, cultivated in the incubator shakers for 30 min under 25 °C (speed 200 rpm/min), then diluted to make suspension (10^8^ spores/ml). A total of 30 healthy *D. helophoroides* adults were placed into the pathogenic fungus suspension for 10 s to allow sufficient contact at the point where they were transferred into clean rearing boxes with sterile forceps. Each strain had three replicates. Samples immersed in the sterile water (Tween-80) were used as control (CK). The artificial diet and moist cotton balls were renewed daily, and their mortality, number of dead individuals, and time of death were recorded. Dead *D. helophoroides* adults were routinely placed into sealed centrifuge tubes (5 ml) inside an artificial incubator (Panasonic, Japan, MIR-154-PC) for 14 d. The incubation conditions were 26 ± 1°C and 80 ± 10% humidity, and colony growth on the surface of *D. helophoroides* adult cadaver was observed daily.

All 17 strains were screened based on the spore production, growth rate, spore germination rate, mortality, cadaver rate, and lethality duration, and the strains with the highest and lowest pathogenicity to *D. helophoroides* adults were determined.

### 2.4. The virulence of the highest pathogenic strains against *D. helophoroides* adults

The strain with the highest pathogenicity was fully eluted with sterile water containing 0.01% Tween-80 in a 50 ml centrifuge tube and fixed to 10 ml, cultivated in the incubator shakers for 30 min under 25°C (speed 200 rpm/min) and then diluted to make 1 × 10^5^, 1 × 10^6^, 1 × 10^7^, 1 × 10^8^, and 1 × 10^9^ spores/ml suspensions. For each suspension, 30 healthy *D. helophoroides* adults were submerged for 5 s before they were placed back into rearing boxes and reared on an artificial diet for 20 d of observation. Sterile water containing 0.01% Tween-80 was used as the control, and each treatment had three replicates.

### 2.5. Biological traits of different strains and identification of highly pathogenic strains

The pure culture of 17 strains was inoculated on the PDA medium, and their spore germination rates were measured. The colony traits were morphologically observed at 24 h, and then their growth rates were measured by the crossover method. Finally, spore production was counted by using a hemocytometer plate after full sporulation at 14 d, and their spore morphology, size, and spore peduncle were also observed and recorded.

DNA from different strains was extracted by using the Ezup column-based fungal genomic DNA extraction kit (Sangon Biotech Co., Ltd., Shanghai, China) and amplified by using the fungal universal primers ITS1 (TCCGTAGGTGAACCTGCGG) and ITS4 (TCCTCCGCTTATTGATATGC). The 25 ul PCR amplification system contained Taq PCE Master Mix 12.5 ul, template DNA 1uL, forward primers, and reverse primers (10 uM) per 1 ul, ddH_2_O 9.5 ul, respectively. The PCR reaction procedure was as follows: pre-denaturation at 95°C for 5 min, denaturation at 94°C for 30 s, annealing at 57°C for 30 s, and extension at 72°C for 90 s for 30 cycles, and finally, all reactions were extended for 10 min at 72°C and detected by electrophoresis using 1% agarose gel. Some PCR samples were specifically selected based on ITS region amplification and sequenced to confirm them as *Beauveria* species. The PCR amplified samples with the ITS region confirmed as *Beauveria bassiana* by electrophoresis were sent to Biotech Bioengineering (Shanghai) Co., for sequencing, then the ITS sequences were submitted to the NCBI database for BLASTN comparison, and 23 fungal sequences were downloaded to construct a phylogenetic evolutionary tree by using the maximum likelihood method (ML) and MEGA10.0 software to determine their species.

### 2.6. Data processing

The cumulative mortality rate [mortality rate (%) = (number of deaths / total number of insects tested) × 100], corrected mortality rate [corrected mortality rate (%) = [(mortality rate in the treatment group–mortality rate in CK) / (1–CK mortality rate)] × 100], and cadaver rate, [cadaver rate, (%) = (number of cadaver / total number of insects tested) × 100] were calculated for each strain. All data were analyzed by using IBM SPSS Statistics 26 software. Figures of cadaver rate and cumulative mortality curve were drawn using Origin 2021b 64 bits. A DSLR camera (Canon EOS 77D) with a macro lens (LAOWA 100 mm Macro) was used to take pictures of the cadavers of *D. helophoroide*.

## 3. Results and analysis

### 3.1. Spore production and virulence of different strains against *D. helophoroides* adults

The spore production of 17 strains varied after 14 d cultivation, of which the BbMQ strain was 1.32 × 10^9^ spores/ml, the spore germination rate was 94.71%, and the colony area was 49.15 mm^2^, which were significantly higher than those of other strains (Table 2, F_18,50_ = 16.931, *p* < 0.05).

**Table 2:** Biological traits of 17 *Beauveria* spp. strains.

**Strain number**	**Growth rate/mm^2^**	**Spore production/ 10^8^ spores/ml**	**Spore germination rate/%**
BbSX-06	20.95 ± 7.56^c^	1.75 ± 1.03^cde^	95.48 ± 1.38^a^
BbMH-03	49.15 ± 5.00^a^	2.77 ± 1.51^cde^	65.38 ± 14.12^d^
BbXP	51.03 ± 10.44^a^	6.37 ± 1.53^b^	91.05 ± 2.79^ab^
BbLJ-06	29.91 ± 4.61^bc^	2.85 ± 1.87^cde^	63.43 ± 9.12^d^
BbQZ-03	12.25 ± 4.22^cd^	0.38 ± 0.20^e^	88.55 ± 5.58^abc^
BbYT-02	17.94 ± 2.52^c^	1.97 ± 0.19^cde^	72.80 ± 10.13^bcd^
BbMQ	49.15 ± 5.01^a^	13.18 ± 0.78^a^	94.71 ± 0.68^a^
BbQZ-08	28.42 ± 6.96^bc^	3.52 ± 0.74^bcde^	80.643.87^abcd^
BbMH-02	40.85 ± 13.56^ab^	0.67 ± 0.35^e^	77.61 ± 8.21^abcd^
BbYT-03	0.00 ± 0.00^d^	0.03 ± 0.03^e^	28.03 ± 11.66^e^
BbDH	1.32 ± 2.92^d^	0.91 ± 0.48^cd^	75.53 ± 1.26^bcd^
BbWYS	25.35 ± 4.52^bc^	4.5 ± 1.87^bcd^	78.36 ± 4.63^abcd^
BbMH-04	31.63 ± 11.75^bc^	3.17 ± 0.93^cde^	66.16 ± 1.13^d^
BbAX-04	30.01 ± 9.23^bc^	2.98 ± 0.5^cde^	74.46 ± 6.39^bcd^
BbMH-01	2.68 ± 4.65^d^	1.58 ± 0.71^cde^	69.65 ± 0.22^cd^
BbHA-03	26.90 ± 6.98^bc^	1.50 ± 1.52^cde^	80.32 ± 2.80^abcd^
BbFD	29.98 ± 8.07^bc^	4.77 ± 1.59^bc^	87.36 ± 6.08^abc^

Data mean ± standard deviation, different lowercase letters after in the same column indicate significance at 0.05 level.

**Figure 1:**
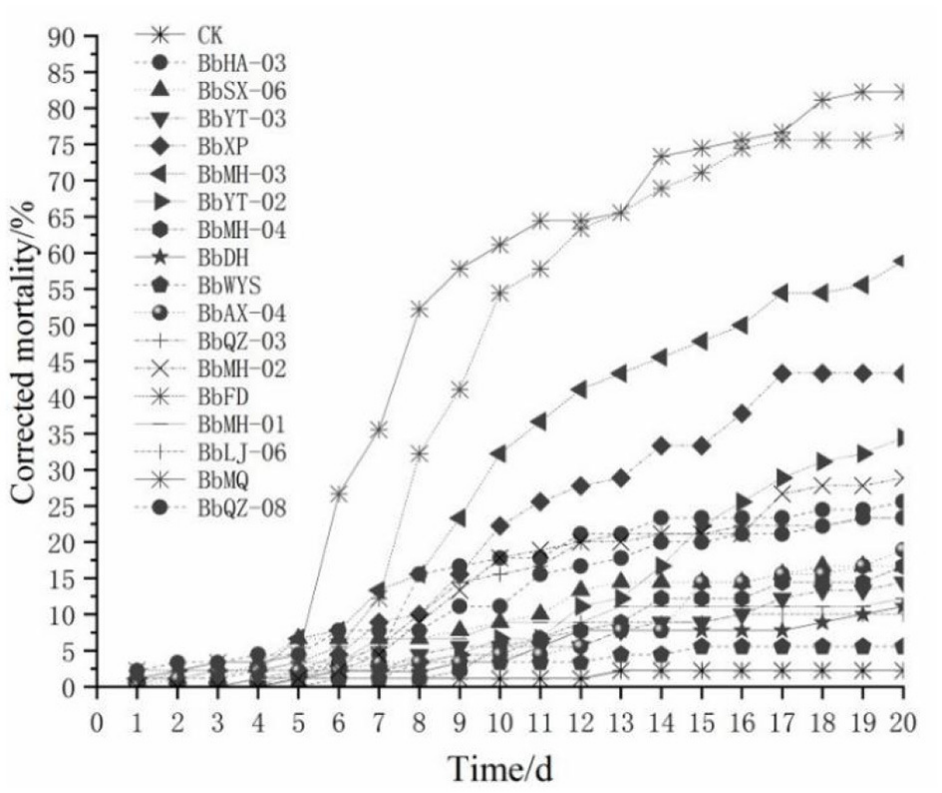
Mortality change of *D. helophoroides* adults infected by different strains of *Beauveria bassiana*.

All tested *Beauveria* spp. were pathogenic to all *D. helophoroides* adults, of which the corrected mortality of the BbMQ strain was 81.82%, indicating that it was the most pathogenic and virulent strain, followed by BbFD strains (76.14%). In addition, the cumulative mortality of the BbMQ strain reached 82.22% at 20 d, which was significantly higher than the other 15 strains (*p* < 0.05). We also found that the mortalities of BbHD, BbWYS, BbMH-01, BbLJ-06, and BbYT-03 strains were <15%, of which the BbWYS strain was only 5.56% (Table 3, F_17,36_ = 11.896, *p* < 0.01). All 17 strains could fit the univariate linear regression equation (F_18,38_ = 12.181, *p* < 0.01, *R*^2^ > 0.85), while the mid-lethal time of the BbMQ strain was theoretically 10.62 d, which was remarkably shorter than the BbFD, BbMH-03 strains (Figure 1, Table 3, F_18,38_ = 12.181, *p* < 0.01).

**Table 3:** Mortality of 17 *Beauveria* spp. strains against *D. helophoroides* adults^①^.

**Strain number**	**Cumulative mortality/%**	**Adjusted mortality/%**	**Regression equation**	**Correlation coefficient**	**Mid-lethal/d**
BbHA-03	25.56 ± 25.56^cd^	23.87^cd^	y = 1.5572x−1.6849	0.8902	33.19
BbSX-06	16.67 ± 0.00^cd^	14.78^cd^	y = 0.9165x+0.2102	0.9534	54.33
BbYT-03	14.45 ± 3.85^cd^	12.51^cd^	y = 0.8128x−2.4786	0.9700	64.57
BbXP	43.33 ± 3.34^bc^	42.04^bc^	y = 2.8044x−8.2799	0.9688	20.78
BbMH-03	58.89 ± 11.7^ab^	57.96^ab^	y = 3.5673x−7.7902	0.9649	16.20
BbYT-02	34.44 ± 18.36^cd^	32.96^cd^	y = 1.899x−7.4406	0.8719	30.25
BbMH-04	16.66 ± 5.77^cd^	14.77^cd^	y = 1.0041x−3.9876	0.9368	53.77
BbDH	11.11 ± 6.94^cd^	9.09^cd^	y = 0.6076x−1.5031	0.9470	84.76
BbWYS	5.56 ± 1.93^d^	3.42^d^	y = 0.3562x−0.6287	0.9323	142.14
BbAX-04	18.89 ± 10.18^cd^	17.05^cd^	y = 0.9785x−2.9975	0.8813	54.16
BbQZ-03	23.33 ± 23.33^cd^	21.59^cd^	y = 1.5312x−2.9113	0.9109	34.56
BbMH-02	28.89 ± 7.70^cd^	27.28^cd^	y = 1.7804x−4.6383	0.9449	30.69
BbFD	76.67 ± 8.82^a^	76.14^a^	y = 5.0529x−9.9448	0.9066	11.86
BbMH-01	12.22 ± 1.92^cd^	10.23^cd^	y = 0.6374x+0.8066	0.9127	79.71
BbLJ-06	10.00 ± 8.82^cd^	7.96^cd^	y = 0.6476x−1.1888	0.9370	79.04
BbMQ	82.22 ± 6.93^a^	81.82^a^	y = 4.9533x−2.6214	0.8941	10.62
BbQZ-08	23.33 ± 6.67^cd^	21.89^cd^	y = 1.2716x−0.1298	0.9699	39.42
CK	2.22 ± 1.92^d^	0.00^d^	y = 0.1335x−0.0701	0.8593	375.06

^①^Data are mean ± standard deviation, different lowercase letters after data in the same column indicate a significant difference at 0.01 level.

### 3.2. Infection symptoms and process of *D. helophoroides* adults by *Beauveria* spp.

The infected *D. helophoroides* adults tended to move slowly and showed slight anorexia during the initial stage of infection. At the beginning of infection (4 ~ 6 d), the number of *D. helophoroides* adults feeding on the diet gradually decreased, and approximately two adults showed daily foraging behavior, and no adults were seen to have fed on the diet during the final period (10 ~ 15 d), while five adults fed daily on the diet in the control groups.

**Figure 2:**
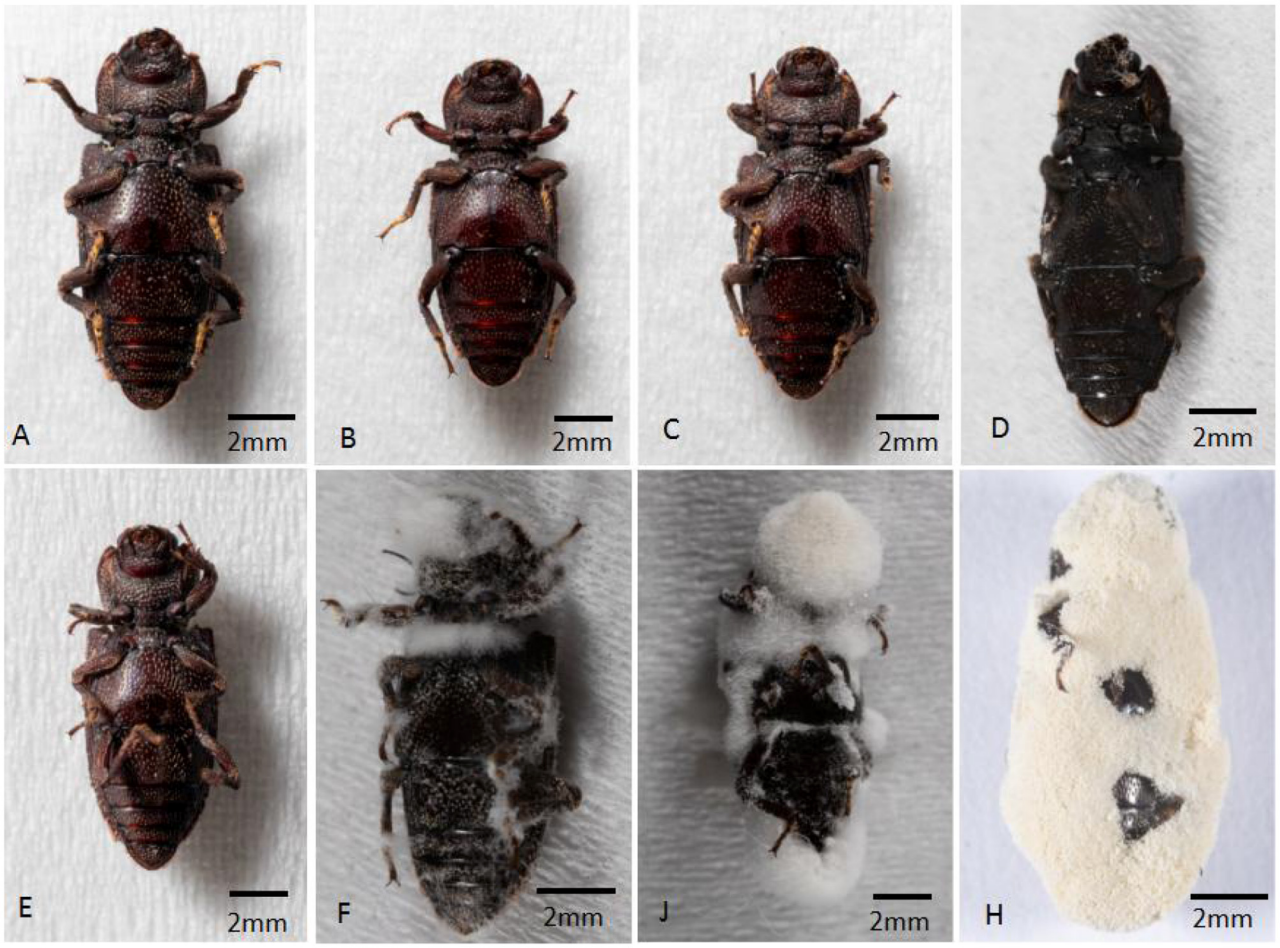
Symptom and process of *D. helophoroides* adults infected by the BbMQ strain. **(A)** 1 d CK; **(B)** 2 d Ck; **(C)** 3 d Ck; **(D)** 11 d CK; **(E)** 1 d BbMQ; **(F)** 2 d BbMQ; **(J)** 3 d BbMQ; **(H)** 11 d BbMQ.

Dead beetles were characterized by having three pairs of legs bent and unfolded after infection, with tarsus clutching the wood, or three pairs of legs stretched out with the abdomen upwards (Figures 2A, E). During 14 d, moisturized culture and the growth of the BbMQ strains was observed as a model: mycelium started from the head mouthparts, thoracic and abdominal ends, and intersegmental areas between each foot of the adult insect after 1 ~ 2 d infection/death, then the whole adult body was covered by the mycelium (Figure 2F), the mycelium grew rapidly on the day 3 (Figure 2J), and a large number of spores started to cover the body surface on day 6 to day 11 (Figure 2H). In contrast, the dead adults from control groups with the moisturizing cultivation did not produce any mycelia and spores during the entire process (0–11 d) (Figures 2B–D).

### 3.3. Cadaver rate of infected *D. helophoroides* adults

**Figure 3:**
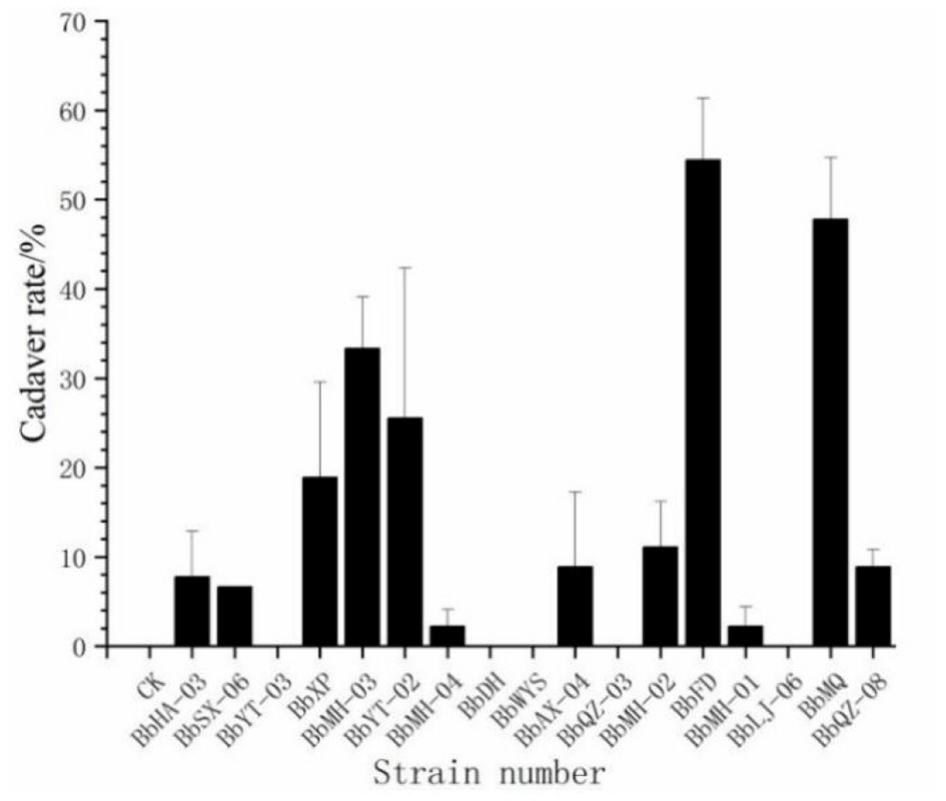
Cadaver rate percentage change of *D. helophoroides* adults infected by different strains of *Beauveria bassiana*.

Out of a total of 17 strains, only 12 strains were found to produce cadavers by moisturizing and culturing the infected carcasses, accounting for 70.6% of all tested strains, and the cadaver rates of BbFD and BbMQ strains were 54.45 and 47.78%, respectively (Figure 3, F_18,38_ = 23.781 *p* < 0.05). The virulence and infestation of *Beauveria* spp. on the *D. helophoroides* adult was relatively stable, and the survival of *D. helophoroides* adults might be seriously threatened by the natural *Beauveria* spp. in the forests.

### 3.4. Virulence of BbMQ strain suspensions

Based on the mortality rate, lethality time, cadaver rate, spore production, spore germination rate, and colony growth area of 17 strains, the BbMQ strain was thought to be the most dangerous to *D. helophoroides* adults. Further results showed that both the cumulative mortality rate and cadaver rate of *D. helophoroides* adults decreased significantly with decreased concentrations of the spore suspension, the lethal time increased significantly with decreasing concentrations of the suspension, and the lethal median time was the shortest (10.88 d) under 1 × 10^9^ spores/ml suspension, indicating that the lethality of the BbMQ strain on the *D. helophoroides* adults was determined by the concentration of the spore suspensions (Table 4, F_5,12_ = 203.738, *p* < 0.05). The semi-lethal concentrations were 7.41 × 10^8^, 4.89 × 10^8^, and 4.43 × 10^8^ spores/ml, respectively, when *D. helophoroides* adults were infected by the BbMQ strain at days 10, 15, and 20, and all fit the univariate linear regression equation (F_4,10_ = 220.038, *p* < 0.05, *R*^2^ > 0.85) (Table 5).

**Table 4:** Pathogenicity of BbMQ strains with different concentrations to *D. helophoroides* adults^①^.

**Concentration /(spores.ml^−1^)**	**Cumulative mortality rate (%)**	**Cadaver rate (%)**	**The regression equation**	**The correlation coefficient**	**Lethal time/d**
1 × 10^9^	92.22 ± 3.85^a^	27.78 ± 12.62^a^	y = 3.5446+11.428	0.7282	10.88
1 × 10^8^	54.45 ± 3.85^b^	31.11 ± 6.94^a^	y = 2.2438x−0.4834	0.8744	22.50
1 × 10^7^	28.89 ± 8.39^c^	16.67 ± 6.67^b^	y = 1.2053x−4.4602	0.9600	45.18
1 × 10^6^	5.56 ± 1.93^d^	2.22 ± 1.92^c^	y = 0.2126x−1.1866	0.8895	240.76
1 × 10^5^	6.67 ± 3.34^d^	0.00	y = 0.2465x−1.7101	0.8725	209.48
CK	0.00	0.00	—	—	—

^①^“—” means that the mortality cannot be linearly fitted with concentrations without dead *D. helophoroides*. Data are mean ± standard deviation, different lowercase letters after data in the same column indicate a significant difference at the 0.05 level.

**Table 5:** Phylogenetic tree of BbMQ strain based on ITS sequence by using Mega10.0 contract.

**Processing time/d**	**Adjusted mortality/%**					**The regression equation**	**The correlation coefficient**	**LC_50_ Lethal median concentration**
	**1**×**10**^6^	**1**×**10**^7^	**1**×**10**^8^	**1**×**10**^9^				
10	1.11 ± 1.92^c^	0.00^c^	5.56 ± 1.93^c^	23.33 ± 5.77^b^	64.44 ± 11.71^a^	y = 0.006x+5.5417	0.9349	7.41 × 10^8^
15	2.22 ± 1.92^d^	2.22 ± 1.92^d^	17.78 ± 1.92^c^	35.55 ± 6.94^b^	87.78 ± 5.09^a^	y = 0.0078x+11.881	0.9070	4.89 × 10^8^
20	2.22 ± 1.92^d^	2.22 ± 1.92^d^	22.22 ± 5.09^c^	44.45 ± 6.94^b^	92.22 ± 3.85^a^	y = 0.0079x+15.023	0.8517	4.43 × 10^8^

^④^Data are mean ± standard deviation, and different lowercase letters after the same data indicate a significant difference at the 0.05 level.

### 3.5. BbMQ strain identification

#### 3.5.1. Morphological identification

**Figure 4:**
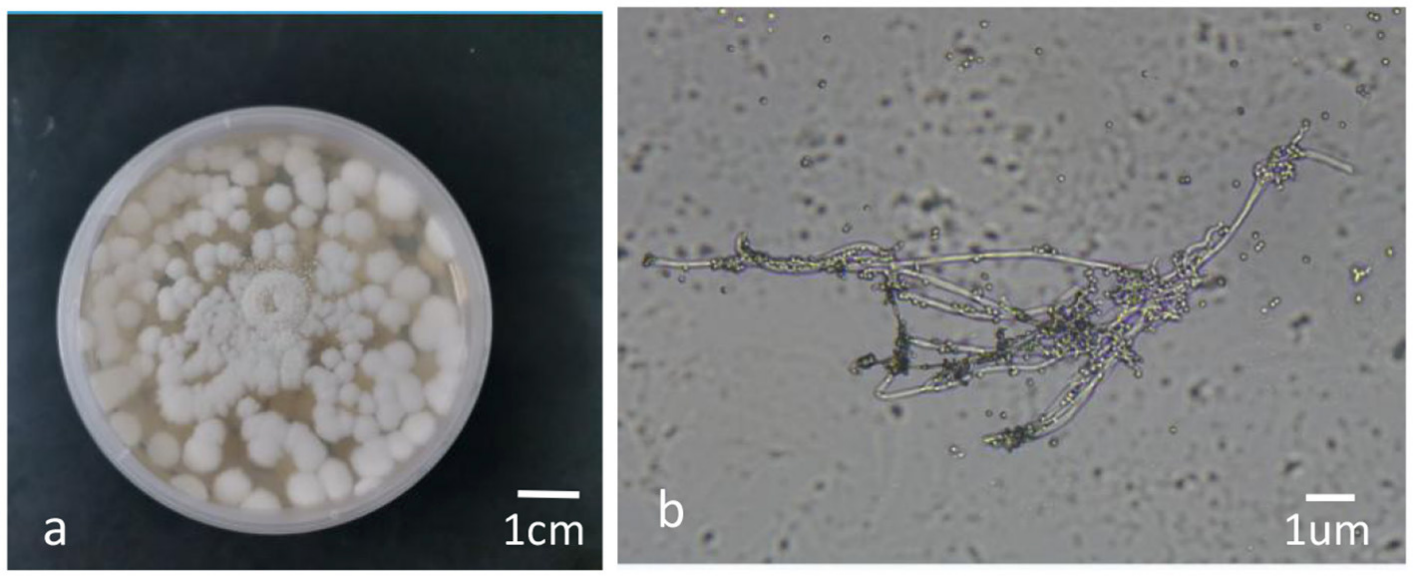
Colony morphology and conidia of BbMQ. **(a)** Colony morphology of the BbMQ strain, **(b)** hypha and conidia of the BbMQ strain.

The colonies of the BbMQ strain were white flocculent at the early stage (0–5 d) and then the mycelium began to produce spores on the day 6 after inoculation. The middle of the colony was white powder-like, the edge was fluffy and neat, and the whole colony was white powder-like on day 8, with a small amount of oil-like droplets on the surface, and the back of the colony was yellowish (Figure 4a). There were some transparent round conidia (Figure 4b) in the sporulation axis extended from the sporulation cell under an optical microscope (NIKON E100) preliminarily indicating *Beauveria bassiana*.

#### 3.5.2. Molecular biology identification

**Figure 5:**
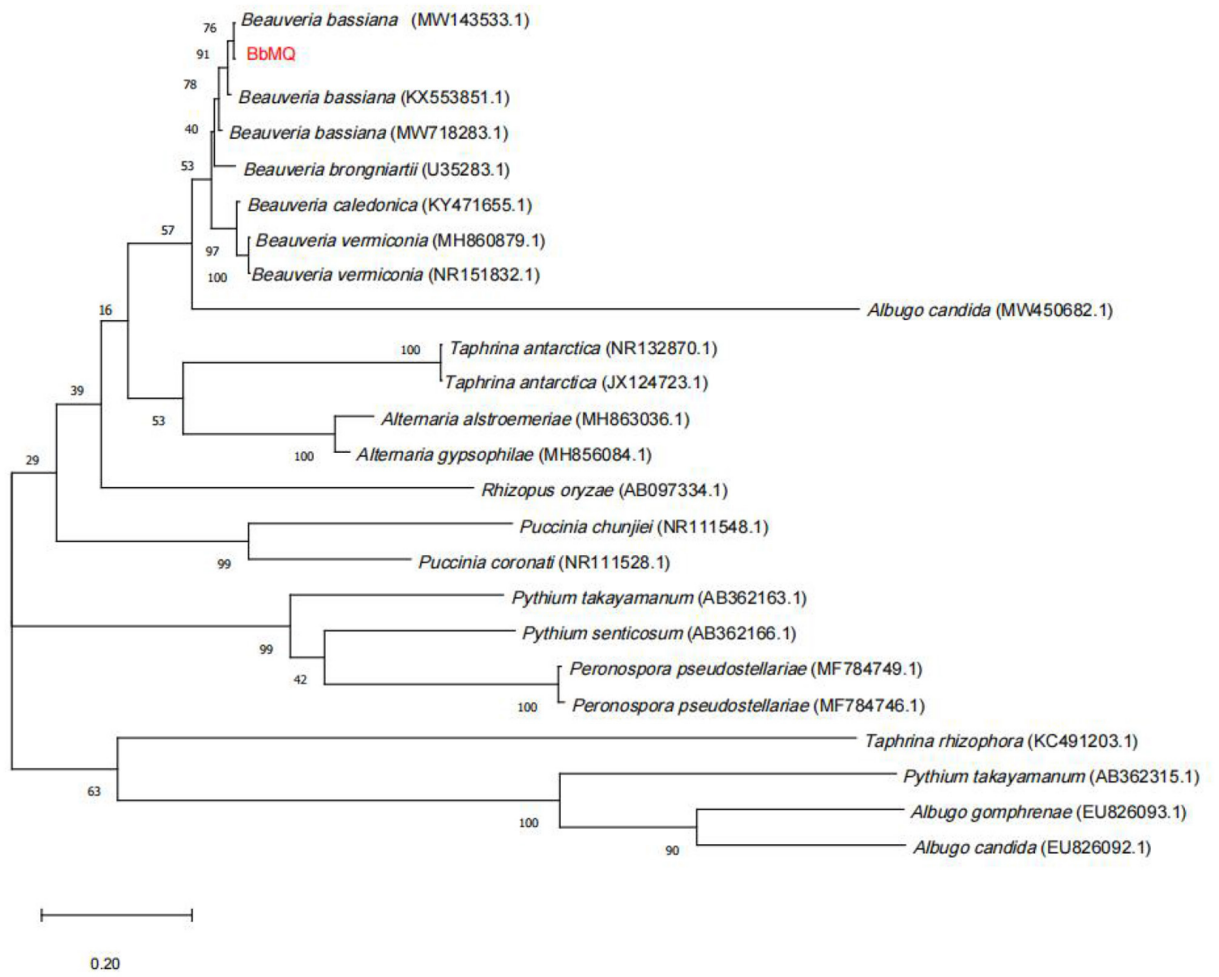
Phylogenetic tree based on ITS sequence by using Mega10.0 contract.

The 533bp amplified sequence was obtained after the BbMQ genomic DNA was used as the template, and the common primers ITS1 and ITS4 were used for amplification, recovery, cloning, and sequencing. The obtained ITS sequences were compared in the NCBI database by BLASTN homology. The sequence homology between BbMQ and *Beauveria bassiana* in the NCBI library was above 99%. In total, 23 sequences with high similarity to *Beauveria bassiana* were selected from NCBI, then a phylogenetic evolutionary tree was constructed using MEGA 10.0 software with a Bootstrap value of 1,000, indicating that BbMQ was in the same sister group as *Beauveria bassiana* (MW143533) strain with high homology (Figure 5). Therefore, the morphology, NCBI blast, and evolutionary tree comparison indicated that the BbMQ strain was *Beauveria bassiana*. The Gen Bank registry of the BbMQ strain was ON386272.

## 4. Conclusion and discussion

*Beauvaria* spp. is the most popular pathogen for some agricultural pests worldwide, especially for defoliators and branch-shoot pests in the forests, and their mass production and technology are mature with promising application prospects (21, 22), easy operation, and bio-control advantages (23). For example, *Beauveria bassiana* was used to control *D. punctatus* (8) and *D. houi* (5) in Fujian, China, achieving maximum mortality rates of 80%, and 92%, respectively. *Beauveria bassiana* from wild strains had a significantly higher lethality than those from commercial strains to control *Scatella tenuicosta* in Texas, USA (24), and mortality of coffee berry borer (CBB, *Hypothenemus hampei*) was more than 90% when *Beauveria bassiana* was applied in the coffee orchard in Hawaii, USA (21).

However, as broad-host insect pathogenic fungus *Beauveria* spp. was widely distributed and persists in various fields worldwide, but it was rarely applied to control *M. alternatus*, (6, 13, 25–27), and its pathogenicity against natural enemies of *M. alternatus* has not been clarified. In this study, we found that 17 strains of *Beauveria bassiana* had significant differences in pathogenicity to *D. helophoroides* adults, with the highly pathogenic strain being BbMQ based on the mortality rate, lethality time, cadaver rate, spore production, spore germination rate, and colony growth dimension. The lethal strain was morphologically and molecular biologically identified as a *Beauveria bassiana* and potentially threatens the safety of *D. helophoroides* adults, while the BbWYS strain had a minimum lethality of only 5.56% but higher lethality (more than 95%) to adults of *Monochamus alternatus* and larvae of *Dendrolimus punctatus*, the most important pests of masson pine forests, which was safer than all other strains to the adult *D. helophoroide* and can be simultaneously released in the pine forests. It should be used cautiously in pine forests to control other pests, such as targeting *D. punctatus* to avoid harming the *D. helophoroides* adults and other natural enemies; meanwhile, the low pathogenic BbWYS strain did not endanger the *D. helophoroides* and would be an ideal alternative natural enemy of controlling other important pests in the pine forests. Additionally, previous studies have shown that *Beauveria bassiana* was highly pathogenic to both adults and larvae of *M. alternatus*, resulting in survival rates of <10%, but mortality was <15% if a higher concentration (1 × 10^8^ spores/ml) suspension was provided to *D. helophoroides* adult and larvae without any cadaver (18), which was significantly different from our findings and presumably related to the pathogenicity differences of various strains.

The combined application of multiple natural enemies can significantly improve the control effect of agricultural and forestry pests. For example, the infected *Bactericera cockerelli* by *Beauveria bassiana* did not have a significant impact on the parasitism of wasps *Tamarixia triozae* (28), indicating that synergistic effects might occur in the pest population when pathogenic fungi and parasitoids were simultaneously released in the forest. The use of *Trichogramma dendrolimi* carrying *Beauveria bassiana* in the field resulted in a 28.1 and 22.8% reduction in the rate of infested *Zea mays* by *Ostrinia furnacalis* and the infesting holes, respectively, and a significant increase in the cadaver rate of *O. furnacalis* (29), while the emergence and survival rate of *T. dendrolimi* was not influenced by *Beauveria bassiana*. An obvious synergistic effect occurred between *Scleroderma guani* and *Beauveria bassiana* when they were released to control the fourth instar overwintering larvae of *M. alternatus* in Lishui County, Jiangsu, China, and the control effect order was *S. guani* plus *Beauveria bassiana* > *S. guani* > *Beauveria bassiana* (30). The combined use of *Scleroderma sichuanensis* and *Beauveria bassiana* was found to cause some damage to *S. sichuanensis*, but it also generally improved the control effect on *M. alternatus* (31).

Physiologically, the spores of different *Beauveria bassiana* strains have different abilities to secrete extracellular enzymes such as chitinase, Pri, and protease to degrade the body wall during the process of breaking through the insect body wall, as well as the amount of secretion, thus showing some infection variability among their hosts (32, 33). We also assumed that differences in their pathogenicity were caused by their different ability to secrete extracellular enzymes.

Overall, the strategy of using natural enemies carrying *Beauveria bassiana* to control agroforestry pests is feasible; however, several concerns need to be addressed. The biosafety assessment of different *Beauveria* spp. strains is necessary prior to their widespread application, which will be beneficial to improve the control effect of forest pests and can also effectively protect some important natural enemies, such as *D. helophoroides*.

## Data availability statement

The datasets presented in this study can be found in online repositories. The names of the repository/repositories and accession number(s) can be found at: NCBI-ON386272.1.

## Author contributions

GL and YZ: conceptualization. GL, YZ, and SY: methodology and writing—original draft preparation. CL, ZC, and XF: software and data analysis. YZ and ZC: validation. YZ, ZZ, and SC: samples collection. YZ, XF, and ZC: investigation. FZ and GL: resources. GL: writing—review and editing and funding acquisition. All authors reviewed and approved the final manuscript.
